# Closing the Loop on the Cocktail Party Effect: From Attention Decoding to Neuro-Steered Selective Hearing

**DOI:** 10.3390/biology15141148

**Published:** 2026-07-14

**Authors:** Qiang Li, Jianmei He

**Affiliations:** 1 College of Education Science, Guizhou Education University, Guiyang 550018, China; hejianmei@gznc.edu.cn; 2 Guizhou Key Laboratory of Artificial Intelligence and Brain-Inspired Computing, Guizhou Education University, Guiyang 550018, China

**Keywords:** auditory attention decoding, cocktail party effect, neuro-steered hearing, selective hearing, closed-loop control, EEG, assistive listening technology

## Abstract

Selective hearing in multi-talker environments remains a central challenge for assistive listening technologies: conventional devices improve audibility but cannot infer which talker the listener intends to follow. This Perspective examines the emerging transition from auditory attention decoding to neuro-steered selective hearing. We argue that a recent real-time intracranial proof-of-principle study marks an important inflection point in this transition, because it shows, under high-fidelity recordings from neurosurgical patients and in controlled two-talker conditions, that decoded neural attention can be coupled online to relative-gain control, thereby improving speech perception and reducing listening effort within that experimental setting. Building on this proof of principle, we argue that the field should move beyond offline decoding accuracy as its primary benchmark and instead treat neuro-steered hearing as a closed-loop translational problem linking neural inference, acoustic scene analysis, and control policy. From this perspective, future benchmarks should integrate device-level control metrics, including latency, effective switch time, false-switch rate and recovery after erroneous updates, with listener-centered outcomes such as speech intelligibility, listening effort, user preference and everyday benefit. Accordingly, we outline a staged roadmap for electroencephalography-based systems, from controlled two-speaker paradigms to scene-aware control and ultimately wearable real-world implementations. We also discuss the complementary roles of magnetoencephalography, functional near-infrared spectroscopy, and functional magnetic resonance imaging in defining the neural mechanisms and design constraints of future systems. Overall, this Perspective frames neuro-steered selective hearing as a systems-level research pathway for evaluating whether neural attention signals can be translated into practical assistive listening technologies.

## Simple Summary

In noisy places, people with hearing difficulties may hear sounds but still struggle to follow one chosen voice. A recent study tested an early way to support this kind of selective listening. In a controlled two-talker experiment, researchers recorded brain activity directly from intracranial electrodes in neurosurgical patients, decoded which talker the listener was attending to, and used that information to adjust the balance between competing speech streams in real time. The system improved intelligibility, reduced listening effort, and was preferred by participants, showing that high-quality neural attention signals can, in principle, guide sound processing to help listeners focus on a target speaker. However, this was an invasive proof of principle, not a wearable or clinically deployable hearing device. This Perspective discusses what must be solved before such ideas could inform future hearing technologies, including non-invasive sensing, reliable control, robustness, device feasibility, and real-world testing.

## 1. Introduction

A longstanding obstacle in assistive hearing is the absence of a reliable mechanism for determining, in real time, which speech stream a listener intends to follow in a multi-talker scene [1,2]. Modern hearing technologies increasingly use directional microphones, adaptive beamforming, scene classification, deep learning-based speech enhancement, and user- or sensor-driven control cues, including head orientation, eye gaze, and audio-visual information, to approximate the listener’s desired target in complex scenes [3,4,5,6]. These advances show that the field is already moving beyond simple amplification toward intent-sensitive and context-aware hearing support. Nevertheless, target selection remains unresolved when multiple plausible speech streams are present, when the intended talker changes over time, or when acoustic, visual, and behavioral cues provide incomplete or conflicting information. This limitation is consequential because difficulty hearing in noise remains one of the central unmet challenges of hearing rehabilitation and is closely tied to reduced communicative participation and social well-being [7,8].

Against this background, auditory attention decoding offers a principled way to add neural evidence to the target-selection problem by transforming neural signatures of selective listening into control signals for adaptive speech enhancement [2,9]. Building on evidence that the auditory system preferentially represents the attended speaker and that selective attention can be decoded from neural recordings, auditory attention decoding has emerged as a plausible route toward hearing technologies that infer the listener’s target talker and adapt dynamically to changing acoustic scenes [9,10,11].

Yet an important limitation has shaped the field. Much of the auditory attention decoding literature has concentrated on discriminating attended from unattended speech, frequently in offline paradigms and with decoding accuracy treated as the primary benchmark [2,9,10]. This body of work has been foundational for establishing the neural and algorithmic feasibility of auditory attention decoding [9,10], but it has left unresolved a more consequential translational question: whether neural decoding can operate with sufficient speed, stability and robustness to support closed-loop acoustic adaptation that yields perceptual benefit in real time [12,13]. In other words, the key issue is no longer only whether attention can be decoded, but whether decoding can be embedded within a control system that improves listening under the conditions in which the cocktail party problem has measurable consequences for real-world listening performance.

A recent study by Choudhari and colleagues [14] addresses this translational gap. In human participants with intracranial recordings, they implemented a real-time closed-loop framework that continuously inferred the attended speaker and used that estimate to regulate the relative gain of competing speech streams. By closing the loop between neural decoding and sensory amplification, the study showed that online neural attention estimates can be coupled to gain control in a way that was associated with improved speech perception, reduced listening effort, and a listening experience that participants generally preferred over the unmodulated condition. The system successfully accommodated both instructed and self-generated attentional switches, indicating that the benefit of neural steering can be sustained as attentional focus changes over time. In a separate validation, listeners with hearing loss evaluated audio that had been modulated by the brain-controlled system and showed improved intelligibility and stronger subjective preference, although they did not themselves provide neural signals or operate the closed loop online. The work therefore establishes, in a controlled intracranial proof-of-principle setting, that decoded auditory attention can be coupled to acoustic gain control to produce measurable selective-listening benefit.

Building on this bounded proof of principle, this Perspective advances a systems-level account of what changes when decoded neural attention is used to control an assistive listening device. The relevant contrast is not between conventional devices that lack control capability and neuro-steered systems that possess it, but between different sources of target-selection evidence—acoustic, visual, behavioral, multimodal, and neural—and the conditions under which neural attention signals may add incremental benefit [15,16]. Its contribution is fourfold. First, it reframes the central performance question from whether attention can be decoded to whether neural evidence can support stable, timely, and perceptually useful closed-loop control. Second, it identifies the acoustic target representation as a critical translational interface: a representation must be not only neurally discriminable, but also manipulable through a meaningful control action such as gain steering, beam steering, or mask-based enhancement. Third, it proposes a staged pathway from predefined laboratory targets to scene-derived candidate objects and, ultimately, wearable real-world operation. Fourth, it specifies an evidence framework in which neuro-steered benefit must be demonstrated relative to strong acoustic-only, gaze-steered, multimodal, and AI-based alternatives, using both device-centered control metrics and listener-centered outcomes. Together, these elements broaden the translational question beyond decoding accuracy and real-time control to include calibration burden, long-term comfort, device miniaturization, embedded resource limits, and the clinical and regulatory evidence needed before eventual deployment. The aim is therefore not to predict that neural steering will necessarily outperform existing technologies, but to define the conditions and experimental tests under which such an advantage could be established.

## 2. A Real-Time Intracranial Proof-of-Principle Demonstration of Neuro-Steered Selective Hearing: Advance and Boundary Conditions

The central advance of Choudhari et al. [14] is to convert auditory attention decoding from a passive readout into an active control signal. Using intracranial recordings from neurosurgical patients, the authors built participant-specific decoders that continuously inferred which of two competing speech streams was attended and then used that estimate to regulate relative gain in real time. In this sense, the study embeds attention decoding within a closed-loop auditory system and uses neural inference as a real-time control signal for selective hearing.

This closed-loop design was not merely conceptually appealing; it produced clear perceptual benefits. When the decoder was allowed to steer amplification online, participants showed improved intelligibility, reduced listening effort, and a clear preference for the system-enabled condition. The decoder also tracked both instructed and self-generated attentional switches, demonstrating that the benefits of neural steering can be maintained even when listening goals change over time. A reverse-gain control further indicated that the direction of the neural-to-acoustic mapping mattered: performance was better when decoded attention and gain updates were aligned, whereas deliberately reversing this mapping degraded perception.

Equally important, the authors showed that the enhanced audio output was beneficial for an independent cohort of listeners with hearing loss. This extends the significance of the work beyond a technically impressive intracranial study. Although intracranial EEG is not itself a scalable assistive platform, the study provides a compelling proof of principle that closed-loop neuro-steered hearing can deliver meaningful benefit, thereby establishing a conceptual foundation for future non-invasive and portable implementations. Read in this way, the paper shows how decoded attention can generate meaningful benefit for selective hearing.

### Boundary Conditions and Limitations of the Choudhari et al. Demonstration

Despite its importance, the Choudhari et al. study [14] should be interpreted as a high-fidelity proof of principle rather than as evidence that a deployable neuro-steered hearing aid is already available. Several boundary conditions are important. First, the real-time closed-loop system relied on intracranial recordings from a small group of neurosurgical participants. Intracranial recordings provide high signal fidelity, focal cortical coverage, and access to spectrally rich neural activity, but these advantages are not available in scalp EEG, ear-centered EEG, or other wearable sensing configurations. Moreover, electrode placement was determined by clinical need rather than by an optimized auditory-attention-decoding montage. Thus, the decoding accuracy and control stability demonstrated with intracranial EEG cannot be assumed to transfer directly to non-invasive or hearing-device-like sensors.

Second, the acoustic environment remained highly constrained. The core closed-loop experiments used a two-talker paradigm in which candidate speech streams were known to the system and could be directly compared with reconstructed neural speech representations. This design was appropriate for testing whether performance depended on the alignment between decoded neural attention and the gain-control action, but it does not solve the more difficult real-world problem of forming, tracking, and enhancing candidate talkers from mixed, reverberant, moving, and multi-speaker acoustic scenes. In deployable systems, errors in source separation, object formation, or target tracking could weaken neural–acoustic correspondence and generate false or unstable gain updates.

Third, the clinical relevance of the hearing-loss validation should be interpreted carefully. The independent listeners with hearing loss evaluated audio that had been modulated by the brain-controlled system, but they did not themselves provide neural signals or operate the closed loop online. The study therefore provides important but bounded evidence that the system-generated acoustic output can be perceptually beneficial for hearing-impaired listeners under those validation conditions. However, it does not establish whether users with different degrees of hearing loss, different ages, or different levels of hearing-device experience would show comparable decoding reliability, calibration tolerance, trust in the control loop, or benefit–risk balance during prolonged use.

Fourth, the study primarily compared the brain-controlled system with system-off and reverse-gain conditions. These controls provide important directional evidence that behavioral benefit depended on the appropriate alignment between decoded attention and gain adjustment. However, they do not by themselves fully isolate the mechanism underlying the observed improvement, because expectation, task strategy, familiarization with the sound environment, adaptation to the gain manipulation, and order-dependent effects could also contribute to intelligibility, effort, or preference differences. Nor do these comparisons determine whether neural information provides incremental benefit over strong acoustic-only, gaze-steered, beamforming, multimodal, deep speech-separation, or AI-based hearing-aid systems operating under the same latency, gain, and output constraints. Future studies will therefore require control-aware benchmarking that combines reverse-gain and system-off comparisons with counterbalanced condition order, adequate familiarization periods, expectation or condition-belief ratings, and, where feasible, fixed-gain, random-gain, yoked-replay, or sham-control variants that match acoustic modulation while removing online neural contingency. Such designs would help separate the specific contribution of online neural-contingent control from broader effects of altered acoustics, expectation, and adaptation. Finally, the use of participant-specific training and smoothed gain control highlights the need to evaluate closed-loop systems not only by decoding accuracy or immediate intelligibility benefit, but also by switch latency, false-switch rate, recovery after erroneous updates, calibration burden, long-term stability, and user acceptance during extended everyday listening.

These limitations do not diminish the conceptual importance of the study. Rather, they define the translational agenda that follows from it. Choudhari et al. [14] provide evidence that correctly aligned neural steering can be associated with perceptual benefit in a tightly controlled closed loop; the next challenge is to determine whether this benefit can be reproduced with non-invasive sensing, scene-derived acoustic targets, uncertainty-aware control policies, strong non-neural comparators, and ecologically valid testing in the intended hearing-impaired user population.

## 3. From Intracranial Proof of Principle to Non-Invasive Neuro-Steered Hearing

Choudhari et al. [14] established a compelling proof of principle for brain-controlled selective hearing: neural estimates of attention can be used online to steer acoustic enhancement and improve listening in multi-talker scenes. The next question is whether analogous closed-loop selective hearing can be achieved with non-invasive modalities, and under what signal and translational constraints. Among these, EEG and MEG are especially informative for capturing the fast cortical dynamics of continuous speech tracking. However, MEG remains limited in portability and real-world deployability, which may constrain its near-term translational use in wearable closed-loop systems. fMRI and fNIRS nonetheless retain substantial value for the field: fMRI offers high spatial specificity, whereas fNIRS provides a more portable optical approach despite its slower hemodynamic signals. These modalities also help identify how attended and unattended speech are differentially represented across cortical regions, thereby clarifying the neural architecture and mechanisms that future neuro-steered systems may exploit. Taken together, EEG currently offers the clearest near-term path toward wearable neuro-steered hearing, while MEG, fNIRS, and fMRI provide complementary temporal, spatial, and mechanistic insight [9,17].

### 3.1. Design Principles and a Staged Translational Roadmap for EEG-Based Closed-Loop Selective Hearing

**Figure 1:**
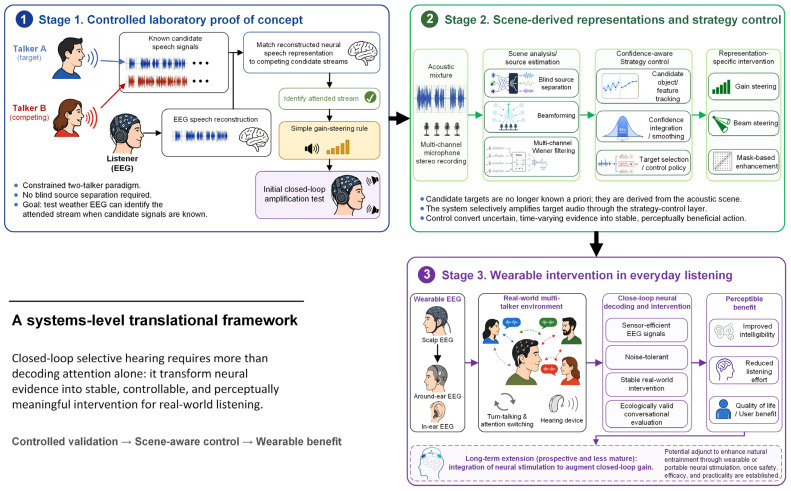
A systems-level translational framework for EEG-based closed-loop neuro-steered selective hearing. Stage 1 establishes proof of concept in a controlled two-talker laboratory paradigm, in which EEG-based speech reconstruction is matched to known candidate speech streams to identify the attended speaker and drive a simple gain-steering rule. Stage 2 extends this framework to realistic acoustic scenes by deriving candidate targets from the acoustic mixture and, when available, from gaze-informed or audio-visual cues through scene analysis and source estimation, and by introducing confidence-aware strategy control that converts uncertain neural evidence into stable, representation-specific interventions, including gain steering, beam steering, and mask-based enhancement. Stage 3 moves toward deployable everyday listening by integrating wearable EEG, real-world multi-talker environments, low-latency closed-loop decoding and intervention, and evaluation in terms of intelligibility, listening effort, and user benefit. Together, the framework outlines a translational path from controlled validation to scene-aware control and ultimately to wearable neuro-steered hearing, with adjunct neural stimulation indicated as a prospective longer-term extension.

Intracranial recordings and scalp EEG capture fundamentally different neural signals and therefore rely on different signal features, representational spaces, and decoding strategies for isolating the attended stream. Intracranial approaches can exploit more focal and spectrally richer cortical activity, whereas scalp EEG provides lower-amplitude, spatially mixed measurements that are more vulnerable to movement, muscle, and environmental artifacts. These fundamental differences, together with the substantial gap in signal-to-noise ratio and spatial resolution, mean that the performance achieved with intracranial recordings cannot be assumed to transfer directly to EEG. Nevertheless, a robust and widely replicated literature shows that scalp-recorded EEG can track the temporal structure of continuous speech, and that this tracking becomes stronger for the speaker to whom the listener is attending in multi-talker environments [18,19]. This attentional modulation provides an independent physiological basis for EEG-based attention-guided auditory control: although its achievable decoding accuracy and control fidelity must be established specifically for scalp EEG, these signals can in principle be used to infer which speech stream a listener intends to follow and thereby support selective-hearing control [10,20]. Figure 1 summarizes this staged translational framework for EEG-based closed-loop neuro-steered selective hearing. Table 1 summarizes the major neural recording modes that may inform neuro-steered selective hearing and compares their translational profiles in terms of latency, decoding reliability, portability, energy demand, computational complexity, and technological maturity.

In practice, this process is typically implemented by reconstructing a speech-related representation from EEG and comparing it with candidate speech streams or acoustic features to determine which signal best matches the listener’s neural response [10]. In this sense, EEG-based attention decoding serves as an interface between neural inference and auditory control: it transforms noisy neural measurements into a confidence-weighted estimate of the attended object, which may subsequently be translated into a control command for downstream auditory enhancement. At this point, the choice of acoustic representation becomes critical because it shapes both decoding accuracy and the form of control that the system can ultimately exert. This dependence is reflected in the diversity of decoding frameworks, which differ not only in predictive performance but also in the representational form through which attention is mapped onto an actionable acoustic target. Approaches based on auditory-inspired subband representations, regularized multichannel lagged decoders, spatial filtering, canonical correlation analysis, and nonlinear short-window models [21,22,23,24,25] therefore expose different forms of controllable output. Stream-like representations are naturally aligned with relative gain control, spatial representations lend themselves to beam steering, and spectrotemporal feature maps are more readily translated into mask-based enhancement. By contrast, more abstract latent features may improve attention inference, but they can influence acoustic control only after they are linked back to a manipulable object such as a stream, mask, or beam.

For closed-loop neuro-steered hearing, decoding methods should be evaluated not only by offline auditory attention decoding accuracy, but also by latency, robustness, computational load, and compatibility with the intended control action. Canonical correlation analysis and regularized linear envelope decoders remain useful low-cost baselines when candidate speech envelopes are available [9,23]. Spatial-filtering and beamforming-coupled approaches are most compelling when sources are spatially separable and acoustic scene analysis is reliable [24]. State-space or Bayesian tracking can stabilize noisy short-window decisions, but this stability is obtained at the cost of additional smoothing delay [12]. Deep neural decoders provide greater representational capacity and can outperform linear baselines in some tasks, but require stronger training data and validation against subject or acoustic-domain mismatch [22,26]. Thus, no single decoder dominates across all deployment criteria; the appropriate choice depends on whether the system prioritizes fast switching, stable gain control, spatial beam steering, or flexible mask/object-level enhancement. Table 2 summarizes the major EEG-based auditory-attention decoding families discussed above, together with their practical conclusions and recommended roles in closed-loop selective hearing.

Building on the preceding discussion of neural recording modalities, EEG-based neural inference, decoding frameworks, acoustic target representations, and representation-specific control actions, we propose three practical design tests for EEG-based neuro-steered selective hearing. First, the neural estimate must identify an attended candidate with sufficient confidence and within a decision window compatible with the intended control action. Second, the candidate must be represented in a form that the acoustic back end can directly manipulate, such that the neural target can be mapped onto an actionable stream, spatial object, or spectrotemporal mask rather than remaining an abstract decoding label. Third, the controller must convert noisy and delayed neural estimates into bounded and reversible acoustic updates, using mechanisms such as confidence thresholds, temporal smoothing, and temporary suspension of neural steering to limit false switches and perceptual disruption. The staged roadmap below tests these dependencies incrementally: Stage 1 isolates neural–acoustic matching using predefined candidate streams; Stage 2 introduces scene-derived targets and uncertainty-aware control; and Stage 3 evaluates whether the complete loop remains beneficial under wearable, computational, and ecologically realistic constraints.

Because the core challenge is to link decoded neural evidence to a stable and manipulable acoustic target, system development is best approached incrementally. The first stage therefore employs simplified two-talker paradigms in which candidate speech streams are known a priori and can serve as predefined targets for control. This controlled setting isolates the neural-acoustic matching problem from the additional complexities of scene analysis, source separation, and object formation. It allows EEG-based reconstruction or decoding to be validated as a reliable indicator of the attended stream. Once this link is established, the inferred target can be coupled to a simple gain-steering rule, providing the most direct proof of principle that EEG-based attention decoding can support minimal closed-loop auditory control.

The second stage removes this simplification: candidate targets are no longer assumed a priori, but must instead be derived from the acoustic mixture itself and maintained in a form that remains both neurally discriminable and operationally useful for control. Closed-loop selective hearing therefore becomes a coupled problem of scene analysis, neural inference, and control. Scene-analysis processing must transform the mixture into candidate streams, spatial objects, spectrotemporal masks, or related features using techniques such as blind source separation, adaptive beamforming, multi-channel Wiener filtering, gaze-informed spatial priors, or audio-visual target-cue integration [4,6,9,27,28,29]. The critical question is whether such representations preserve sufficient attention-relevant structure to support reliable EEG-based target inference while remaining actionable for downstream auditory enhancement. This requirement is especially demanding because the candidate representations are themselves only imperfect estimates of the underlying scene: separation errors, residual noise, and reverberation can blur object boundaries, weaken neural-acoustic correspondence, and destabilize the control decisions built upon them, particularly for envelope-based approaches when speaker-specific envelopes cannot be robustly recovered [30]. Under these conditions, neural decoding cannot directly drive enhancement in a frame-by-frame manner, but must be mediated by a strategy-control layer that absorbs uncertainty and converts probabilistic neural evidence into stable intervention. This intermediate layer tracks candidate objects or features over time, estimates the most likely attended target, integrates temporal smoothing and confidence metrics, and determines how inferred attention should modify the acoustic scene. Crucially, the available control actions depend on the form of the underlying representation: separated streams support relative gain adjustment, spectrotemporal masks enable selective enhancement and suppression, and spatial representations lend themselves to beam steering, whereas more abstract latent features contribute only indirectly unless they can be mapped back onto a manipulable acoustic object. Recent foundation models and multimodal architectures may also reshape how candidate targets are represented in closed-loop selective hearing. Rather than treating candidate targets only as envelopes, separated streams, or spatial estimates, or gaze-indexed audio-visual objects, future systems may derive neurally testable scene hypotheses from large-scale pretrained models such as Whisper, BEATs, CLAP, and AV-HuBERT, which encode speech content, speaker characteristics, acoustic events, and, where available, visual speech cues [31,32,33,34]. In this setting, neural decoding would not be replaced; rather, its role would shift toward selecting among richer and more semantically structured candidate objects. This shift could make closed-loop selective hearing less dependent on low-level envelope matching alone and more compatible with realistic conversational settings, including multilingual speech, partial target observability, and face-to-face interaction in which eye gaze and visual speech information is informative [31,34]. By explicitly linking scene-derived representations, neural inference, and representation-specific control policies, the system ensures that decoding leads to stable and perceptually meaningful auditory intervention rather than remaining an isolated inference step.

Finally, a third stage targets deployable neuro-steered hearing systems for everyday listening. At this stage, translational progress depends on stable operation under realistic constraints of wearability, user comfort, calibration burden, device miniaturization, latency, computational budget, energy availability, thermal safety, ecological variability, and clinical/regulatory evaluation. Wearable sensing becomes central to the roadmap. Around-ear and in-ear electroencephalography support prolonged and unobtrusive use, but available evidence shows a clear trade-off between wearability and decoding performance, especially when decisions must be made over short windows [35,36]. These constraints make wearable neuro-steered hearing a problem of hardware–algorithm–user co-design and clinical/regulatory planning rather than decoder portability alone.

Latency budget should be specified at the full-system level. The acoustic input-to-output path must remain within conventional hearing-device constraints and should not be slowed by neural decoding; hearing-aid delay studies indicate that delays of approximately 20–30 ms can become disturbing, while open-fit simulations show that even differences within the 0.5–10 ms range can affect preferred sound quality [37,38,39]. By contrast, the neural-control path can operate on a slower timescale because it updates gain, mask, or beam settings asynchronously rather than rendering the acoustic waveform sample by sample. For this path, the key metric is the effective attention-to-control switch time, consistent with switch-duration metrics proposed for neuro-steered gain control [2]. Existing real-time work places this quantity in the seconds range: Bayesian M/EEG attention tracking has used fixed-lag smoothing with approximately 1.5–1.9 s built-in delay and additional transition delay [12], whereas the intracranial closed-loop system of Choudhari et al. [14] used a 4 s decoding window with 0.5 s updates and reported a mean instructed-switch time of 5.1 s, explicitly attributing this latency mainly to stability-oriented design choices rather than to a neurophysiological limit [14]. Thus, rapid conversational steering should aim for effective switches within a few seconds, while values around or above 5 s should be justified as slower scene-level adaptation rather than rapid talker switching. Future studies should therefore report the neural evidence window, update interval, computation time, smoothing delay, acoustic rendering delay, effective switch time, false-switch rate, and embedded power and thermal costs.

Delay-related benefit should also be evaluated in terms of speech intelligibility rather than subjective sound quality alone. Recent open-fit hearing-aid simulations showed that processing delays in the 3–10 ms range can worsen the speech reception threshold (SRT) in unilateral open-fit provision when speech and noise are spatially separated, whereas comparable bilateral open-fit simulations showed no significant delay-dependent SRT change [40]. This indicates that delay effects are configuration-dependent rather than governed by a single universal threshold: intelligibility loss is most likely when delayed processed sound disrupts binaural timing cues or mixes asymmetrically with direct sound. Model complexity should be interpreted in the same platform-dependent way. There is no universal parameter-count upper limit for embedded deployment; feasibility is determined jointly by algorithmic delay, computation latency, memory and data movement, numerical precision, power consumption, and thermal constraints. Hearing-aid-oriented neural enhancement studies illustrate this operating envelope: compressed and quantized recurrent models have achieved millisecond-scale computational latency within a 10 ms processing target [41], while recent real-time multichannel and embedded field-programmable gate array studies show that low-complexity design, on-chip memory access, and precision reduction can determine whether enhancement remains within a few-millisecond to 10 ms latency range [42,43]. These examples suggest that claims of embedded feasibility should be tied to a declared platform and processing target, rather than inferred from parameter count alone. Table 3 summarizes the major neural and non-neural evidence streams discussed above, emphasizing their current strengths, translational constraints, and appropriate benchmarking roles for closed-loop selective-hearing control.

From a computational perspective, a closed-loop system may need to perform several demanding operations in parallel or near-parallel: continuous multi-microphone signal capture, scene analysis and candidate-signal formation, speech separation or beamforming, EEG preprocessing and artifact suppression, neural decoding or neural-acoustic matching, confidence estimation and temporal smoothing, and finally the control update that adjusts gain, masks, or beam direction. Many of these operations can currently be supported by desktop-class central processing units, graphics processing units, or external workstations in laboratory settings, but a wearable device cannot assume that level of computational headroom while also meeting strict limits on size, battery capacity, memory, and heat dissipation. Replicating even part of the functionality currently delivered by desktop- or server-class pipelines on a wearable platform will therefore require substantial advances in embedded computation, achieved not only through hardware miniaturization but also through algorithms designed around bounded memory, bounded latency, and reduced arithmetic intensity. In practice, this will require algorithmic strategies that reduce memory use and arithmetic cost under the latency and data-access constraints of wearable operation. For example, recursive time-adaptive stimulus-reconstruction decoders can achieve performance comparable to sliding-window implementations while requiring substantially less memory and lower computational cost [44], whereas real-time attention-tracking frameworks based on online model estimation and Bayesian fixed-lag smoothing can approximate batch-mode state estimation in near real time, with limited data access, controllable delay, and lower computational complexity [12]. More generally, wearable deployment may require compact neural representations, low-complexity candidate generation, model compression or quantization, cascaded or event-triggered inference, sparse or approximate computation, and other lightweight decoding or control strategies that reduce computational demand without unacceptable loss of performance.

A complementary systems-level response to these computational and resource constraints is to distribute computation across the hearing device and a companion platform. In such an architecture, time-critical sensing and control can remain on the hearing device, whereas computationally heavier processing, longer-timescale state estimation, and user-facing functions can be assigned to a smartphone or similar companion device with greater available compute, memory, and interface capacity. In such an architecture, thermal load should also be treated as a primary translational constraint, because processor utilization, battery discharge, and enclosure design may all generate heat near the ear or scalp, potentially compromising comfort, reliability, and prolonged wear. Accordingly, distributed architectures should be evaluated by how they allocate latency-critical and resource-intensive functions across the hearing device and companion platform, and by whether this allocation improves battery life, thermal comfort, and control stability during prolonged use. Beyond engineering performance, validation should also rely on ecologically grounded paradigms that incorporate free movement, conversational turn-taking, endogenous attention switches, and hearing-impaired listeners, because these conditions determine whether selective cortical tracking remains sufficiently informative in dynamic natural listening [45,46,47].

Although the most immediate translational pathway remains improved acoustic control, a longer-term extension may involve adjunct neural interventions. For example, speech-envelope-shaped transcranial stimulation could in principle complement acoustic steering, although such approaches remain largely experimental at present [48,49]. Taken together, this stage advances electroencephalography-based selective hearing not toward an immediately wearable solution, but toward a resource-constrained, computation-limited, low-latency, and behaviorally validated assistive system whose feasibility will depend on explicit hardware, algorithmic, embedded-computing, energy, and thermal co-design.

In summary, EEG-based closed-loop selective hearing is best viewed as a staged systems-level translational framework in which neural speech tracking provides the physiological basis for control, but does not by itself guarantee functional selective hearing. The key challenge is to transform decoded attentional evidence into stable and perceptually meaningful auditory intervention by linking neural inference to an appropriate acoustic representation and a corresponding control policy. Because different representations afford different forms of manipulation, progress depends on jointly improving decoding accuracy and ensuring that neural targets remain both discriminable and actionable across increasingly realistic listening conditions. A coherent translational pathway therefore moves from simplified paradigms with predefined streams, to scene-derived objects governed by confidence-aware strategy control, and finally to wearable, low-latency, ecologically validated systems for everyday use.

### 3.2. Complementary Roles of MEG, fNIRS, and fMRI in Non-Invasive Neuro-Steered Hearing Research

MEG plays a complementary role in non-invasive neuro-steered hearing research because it can resolve the timing and cortical sources of selective speech processing with greater spatial specificity than conventional scalp EEG. Studies of multi-talker listening indicate that relatively early auditory responses retain a mixture-like representation of the acoustic scene, whereas later responses increasingly emphasize the attended stream as a segregated auditory object and, at subsequent stages, as a higher-order linguistic representation [50,51,52]. MEG is therefore particularly useful for identifying candidate response latencies, cortical regions, and representational levels that may contain attention-related information. These findings should, however, be treated as hypotheses and candidate priors for decoder development rather than as evidence that mechanistic resolution alone improves a deployable auditory-attention-decoding system.

Historically, the translation of MEG findings into measurable decoding improvements has been partial but not absent. MEG evidence that attended speech produces stronger envelope-related neural tracking and attention-sensitive temporal-response-function components, including the M100 response, directly motivated biophysically informed state-space decoders [50,53]. Akram et al. used these properties to obtain probabilistic attention estimates with confidence bounds at a temporal resolution of seconds, reporting improvements in temporal resolution, computational complexity, and decoding accuracy relative to earlier approaches [53]. Miran et al. subsequently incorporated MEG-derived M100 and stimulus–response markers into a near-real-time M/EEG Bayesian tracking framework, demonstrating approximately 2 s built-in delay and measurable attention-tracking performance under controlled two-speaker conditions [12]. These studies constitute evidence of translation from MEG-derived neural markers to decoder design and dynamic state estimation [12,53]. However, later MEG findings concerning source-resolved cortical hierarchy, object-based alpha activity, and linguistic representations have mainly established neural decodability or behavioral relevance, rather than consistent superiority over conventional sensor-space or envelope-based auditory-attention decoders under matched conditions [51,52,54]. It also remains unclear whether MEG-derived source, latency, or feature priors improve scalp or ear-centered EEG decoding, reduce calibration burden, shorten decision windows, or produce greater closed-loop perceptual benefit. MEG should therefore be framed as a mature platform for biomarker discovery, algorithm development, and mechanistic validation, while its cross-modal and device-level translational value remains to be demonstrated experimentally.

fNIRS may offer a distinctive contribution to closed-loop neuro-steered hearing, particularly when selective listening needs to be studied under conditions that more closely resemble natural face-to-face communication. Compared with fMRI, fNIRS is quiet, portable, and compatible with relatively unconstrained interaction, while still providing a degree of spatial sensitivity to cortical hemodynamic activity. Relative to EEG, fNIRS can also provide more spatially interpretable information about cortical regions engaged during selective listening, which is useful for linking attention-state estimates to the regional organization of speech processing. This is especially relevant because selective speech listening is not mediated by a single neural response, but involves hierarchically organized and functionally differentiated cortical regions that respond differently to attended and unattended speech streams [11,55]. In naturalistic multi-speaker settings, fNIRS has shown stronger synchronization with the attended than the unattended speaker in sensorimotor and temporal regions, suggesting that hemodynamic activity can capture sustained neural signatures of selective listening [56]. Recent dichotic-listening work has further shown that auditory attentional targets can be discriminated from fNIRS activation patterns with accuracies of up to 73.7%, supporting the feasibility of fNIRS-based attention-state estimation under controlled conditions [57]. These findings indicate that fNIRS may be particularly valuable for identifying cortical regions involved in speech selection and for tracking sustained attentional states over multi-second timescales. Because the hemodynamic response evolves more slowly than electrophysiological signals, fNIRS is unlikely to replace EEG as the primary modality for real-time neuro-steering. At the current stage, its value is better framed as a complementary research tool for clarifying the cortical organization and sustained hemodynamic correlates of selective listening, thereby informing the interpretation, validation, and future refinement of EEG-dominant or hybrid neuro-steered hearing frameworks.

fMRI occupies a different role in non-invasive neuro-steered hearing research. It is poorly suited to near-term closed-loop selective-hearing applications because scanner-based acquisition is non-portable and the scanner itself introduces substantial acoustic noise. Its main value instead lies in spatial specificity: fMRI can localize, at the whole-brain level, where attentional modulation emerges across the speech-processing hierarchy and how attended and unattended speech are differentially represented across cortical regions and representational levels. In this sense, fMRI is best understood as a theory-building and model-constraining tool. By identifying the cortical loci and representational stages most relevant to selective listening, it can inform the interpretation of EEG and fNIRS signals, guide sensor placement and target selection, and motivate proof-of-concept closed-loop studies that compare the utility of control signals derived from different brain regions.

## 4. Standardized Evaluation and Research Priorities for the Next Phase of Neuro-Steered Selective Hearing

The next phase of neuro-steered selective-hearing research should ask a more practical question: under what conditions can closed-loop neural steering provide measurable benefit beyond existing assistive strategies? The central task is therefore to determine the conditions under which auditory attention decoding can support stable, timely, and perceptually beneficial control, and to convert the major translational obstacles identified above into testable research tasks. Table 4 maps these challenges to concrete design recommendations and evaluation endpoints. Its purpose is not to imply that a deployable neuro-steered hearing aid is already within reach, but to specify how decoder performance, latency, acoustic target formation, wearable sensing, calibration burden, user comfort, uncertainty handling, device miniaturization, embedded feasibility, and clinical or regulatory readiness can be studied within a coordinated experimental framework.

### 4.1. Toward Tiered Benchmarking Frameworks and Benchmark Resources

A standardized evaluation framework is needed to prevent neuro-steered hearing from being judged by a single metric such as offline decoding accuracy. At minimum, future studies should distinguish three levels of evaluation: first, offline auditory-attention decoding on shared datasets with rigorous train-test separation; second, replay-based or simulated closed-loop testing in which the same acoustic input, enhancement backend, gain limits, and latency budget are held constant while the control signal is varied; and third, online user testing in which closed-loop metrics are combined with perceptual and experiential outcomes. Across these levels, studies should report not only decoding accuracy, but also neural evidence window, update interval, computation time, acoustic input-output delay, effective attention-to-control switch time, false-switch rate, missed-switch rate, recovery after erroneous updates, speech intelligibility or speech reception threshold, validated listening-effort measures, preference, calibration time, and everyday user-reported benefit.

Public datasets can support the first level of this benchmark, although no single existing dataset captures the full closed-loop, wearable, and hearing-impaired use case. Representative resources include the DTU EEG-and-audio AAD dataset [58], which contains EEG recordings from 18 normal-hearing listeners attending to one of two competing speech streams under different reverberation conditions; the KU Leuven AAD dataset [25], a widely used benchmark that also highlights the need for rigorous cross-validation and control of trial- and gaze-related biases; the AV-GC-AAD dataset [59], which was designed to disentangle auditory attention from eye-gaze shortcuts; the DTU normal-hearing/hearing-impaired dataset [60], which includes both EEG and behavioral measures from 22 hearing-impaired and 22 normal-hearing participants; and emerging ear-EEG or spontaneous-switching datasets [36] that are useful for testing wearable sensing and dynamic attention shifts. These datasets should be treated as complementary benchmark tiers rather than as interchangeable validation resources, because they differ in participant population, sensor configuration, acoustic realism, availability of behavioral outcomes, and relevance to online control.

### 4.2. Demonstrating Incremental Benefit: Comparator Design and Listener-Centered Outcomes

Within this benchmark architecture, the decisive translational question is not whether a neuro-steered system improves listening relative to unprocessed or weakly processed audio, but whether neural attention information provides incremental benefit beyond strong acoustic-only and AI-based assistive systems. Adaptive beamforming, deep speech separation, and modern deep learning-based hearing-aid processing should therefore be treated as active comparators rather than as generic conventional baselines. A fair evaluation should hold the acoustic input, microphone configuration, acoustic back end, output-gain limits, and latency budget constant, and should vary the source-selection or control signal across acoustic-only, non-neural intent-cue, neural target-selection, and oracle-target conditions where feasible. This design would help determine whether any observed benefit arises from neural information itself rather than from a stronger downstream enhancement algorithm.

Although decoding accuracy has been valuable for characterizing system performance, the next stage of the field will benefit from benchmarks that more directly capture how a closed-loop device operates in real time. Future studies should report closed-loop performance measures such as command-update delay, effective switch time following an attentional shift, false-switch rate, recovery time after an erroneous update, and the cumulative perceptual benefit delivered during continuous listening. These measures should be assessed at the level of the full control loop, encompassing scene analysis, target tracking, decision smoothing, and acoustic intervention. A major methodological priority is to identify the performance measures that best predict whether users experience more stable and useful listening.

For this reason, device-centered control performance should be paired with listener-centered outcomes rather than interpreted as sufficient evidence of user benefit. Improved intelligibility and user preference are important, but they do not by themselves demonstrate that a system reduces the cognitive burden of listening. Listening effort is commonly treated as a multidimensional construct that reflects the cognitive resources required to achieve successful communication, particularly under challenging listening conditions [61,62]. Future benchmarks should therefore combine closed-loop control metrics with validated effort ratings, behavioral measures such as response speed, task accuracy, or secondary-task performance under comparable intelligibility conditions, and physiological markers such as task-evoked pupillary responses [63]. These outcomes should also be linked to clinically meaningful evidence, including speech-in-noise performance and everyday user-reported benefit. This combined framework would help determine not only whether neuro-steered hearing systems update control commands accurately, but also whether those updates make communication easier, less demanding, and more useful in daily life.

### 4.3. Scene-Derived Target Formation and Uncertainty-Aware Control

A further priority is to compare acoustic target representations as control substrates, because the choice of representation shapes both the decoding problem and the form of downstream control. Stream-based targets, spatial targets, and spectrotemporal masks do not merely provide different inputs to a decoder; they define different forms of downstream control and therefore different failure modes. Future work should directly compare these representation classes within matched acoustic scenes and matched neural-inference pipelines. Such studies could help determine whether translational utility aligns more closely with attention classification accuracy, control stability, or perceptual benefit after enhancement. This question is relevant because a representation that is neurally discriminable may still be poorly suited to intervention if it cannot be manipulated robustly in adverse acoustic scenes.

Uncertainty handling and control policy central objects of study. In realistic environments, neural evidence will often be noisy, delayed, or ambiguous, and scene-derived targets will frequently be imperfect. These conditions make uncertainty a governing factor in system behavior, shaping when interventions are introduced, how rapidly control adapts, how strongly candidate targets are weighted, and how the system revises its behavior as evidence evolves over time. Closed-loop auditory attention systems are therefore best treated as sequential decision systems whose performance depends on how they manage responsiveness, stability, reversibility, and the perceptual consequences of control errors under changing acoustic and behavioral conditions. A useful research direction is to identify policy architectures and uncertainty-management strategies that preserve perceptual continuity, remain responsive to genuine attentional shifts, and degrade gracefully when neural or acoustic estimates are unreliable. Under these conditions, the control layer should be able to down-weight, delay, or temporarily suspend neural steering when neural evidence becomes unreliable, rather than forcing an erroneous update.

### 4.4. Foundation-Model and Multimodal Integration for Scene Analysis and Control

A related priority is to determine how foundation models and multimodal architectures should be integrated into neuro-steered hearing in ways that improve scene analysis, control, and interaction. One likely contribution is at the stage of candidate formation: large pretrained audio encoders such as Whisper, BEATs, and CLAP, together with audio-visual models such as AV-HuBERT when visual speech cues are available, may help replace fragile low-level target representations with richer speech-, speaker-, and event-aware scene hypotheses, provided that these hypotheses remain neurally discriminable and mappable onto controllable objects such as streams, masks, or spatial targets [31,32,33,34]. A second contribution may lie in control and user interaction: audio-language and multimodal models, including systems such as SALMONN and SeamlessM4T, could support semantic scene parsing, multilingual interaction, user-query grounding, and longer-timescale decisions about when neural evidence should trigger gain control, beam steering, captioning, translation, or requests for user confirmation. On the neural side, emerging EEG foundation models such as LaBraM and NeuroLM suggest that pretraining across heterogeneous datasets may reduce calibration burden, improve cross-session generalization, and support more flexible decoding interfaces. In this view, large language models would be less likely to replace auditory attention decoding than to extend the scene-analysis, control, and user-interaction components around it. The resulting paradigm would therefore differ from classical auditory attention decoding in an important way: neural signals would no longer be used only to choose between a small number of predefined streams, but to disambiguate listener intent within a richer multimodal model of the acoustic scene and the user’s communicative goal. This shift, however, also introduces substantial translational constraints. In addition to latency, privacy, and hallucination risk, practical deployment in hearing devices will be shaped by the limited compute capacity, memory, battery life, and thermal envelope of embedded platforms, which may require compression, distillation, cascading, or selective offloading of model components. Future studies should therefore benchmark foundation-model-assisted and classical pipelines against a broader set of criteria, including closed-loop benefit, controllability, computational efficiency, and robustness in real-world conversational settings.

### 4.5. Wearable Translation: Personalization, Long-Term Robustness, and Device Feasibility

Wearable translation also depends on inter-subject personalization, calibration burden, generalization, and longitudinal robustness. This issue is central to translation because neuro-steered systems must accommodate substantial across-user variability in cortical speech-tracking strength, response latency, scalp or ear-centered signal topography, hearing status, and attentional strategy, while also remaining stable despite head movement, muscle artifacts, inter-electrode and inter-user contact-impedance mismatch, electrode-impedance drift, and prolonged use under minimal recalibration. Personalization should therefore be treated as a core design requirement rather than a post hoc fitting step. Future systems will likely need hybrid learning strategies that combine population-level or pretrained models with lightweight user-specific adaptation, brief initial calibration, online self-supervised updating, and event-triggered recalibration when signal quality, contact quality, channel reliability, or decoding confidence deteriorates. Addressing these challenges will require a layered robustness strategy spanning sensor mechanics, online artifact suppression, impedance-aware interface monitoring, subject-adaptive decoding, and cross-session adaptive decoding. Head-movement sensitivity should first be reduced at the sensor interface by using mechanically stable ear-centered or auricular-conformal electrodes, active front ends, short and well-stabilized interconnects, and, where feasible, auxiliary motion or noise-reference sensing [64]. Residual movement contamination can then be handled through real-time-capable denoising approaches such as adaptive filtering, artifact-subspace methods, or dual-layer/noise-reference regression [65]. Muscle artifacts, especially those arising from jaw motion, facial activity, and neck tension during natural conversation, require a parallel strategy: real-time suppression based on canonical correlation analysis is best combined, where feasible, with reference-assisted source-separation approaches that incorporate auxiliary surface electromyography channels from peri-auricular or neck muscles [66,67,68,69]. Because jaw-related contamination is particularly prominent in ear-centered recordings and myogenic activity overlaps strongly with higher-frequency EEG content, these suppression steps should be complemented by real-time contamination detectors [70,71]. When muscle and movement artifacts co-occur in mobile listening conditions, broader online cleaning frameworks may provide an additional safeguard. Variations in electrode impedance during extended wear should likewise be treated as an online systems problem, addressed through high-input-impedance active electrodes, continuous contact-quality or impedance monitoring, and event-triggered recalibration instead of repeated full-session recalibration. Over longer timescales, stable operation will also depend on soft and conformal dry interfaces, skin-compatible materials that preserve comfort and contact stability during extended wear, anatomically individualized ear-centered designs, and decoders that can adapt across sessions and sparse wearable montages with limited labeled recalibration. In this sense, robustness to movement, muscle activity, interface drift, and prolonged use is best framed as a closed-loop co-design problem spanning materials, sensor mechanics, online denoising, adaptive decoding, and confidence-aware control. This places methodological emphasis on learning strategies that remain stable under session-to-session variability, tolerate sensor displacement, and adapt with little or no subject-specific retraining. Future studies should therefore move beyond single-session optimization to characterize cross-session performance, calibration time, and placement sensitivity, while testing approaches such as session-invariant representations, sparse-sensor decoding, and few-shot or unsupervised recalibration.

Beyond personalization and longitudinal robustness, device-level feasibility should be tested as part of the closed-loop system itself. For wearable neuro-steered hearing, an algorithm that performs well in offline or workstation-based settings may still fail translation if it exceeds the thermal, memory, battery, or reliability envelope of an ear-level device. These device-level constraints are especially consequential in wearable implementations because they directly affect comfort, operational robustness, and sustained usability. In this context, recent biomedical-device research suggests that integrated finite element method and artificial intelligence approaches may provide a useful methodological route: finite-element models can characterize heat transfer, stress distributions, and device-specific operating limits, whereas artificial intelligence models can serve as fast simulation surrogates, support hotspot or fault-state classification, and accelerate the screening of alternative designs across large parameter spaces [72,73]. Incorporating such methods into neuro-steered selective-hearing research would help extend evaluation beyond algorithmic performance alone and clarify which system configurations are most likely to support safe, reliable, and computationally sustainable closed-loop benefit in real-world wearable use.

### 4.6. Adverse User Experience, Clinical Heterogeneity, and Human-Factors Evaluation

A further translational priority is to treat adverse user experience as a primary outcome domain. In a neuro-steered selective-hearing system, incorrect or delayed control may amplify the wrong talker, suppress the intended talker, destabilize the auditory scene, or delay adaptation after an endogenous attentional shift. These events are likely to be experienced as perceptual disruption rather than as abstract system error, and may manifest as annoyance, subjective discomfort, reduced trust, increased manual correction, or withdrawal from device use. The bounded proof-of-principle study by Choudhari et al. [14] already suggests that such error states are perceptually meaningful: lower trial-level decoding fidelity reduced preference for the system-enabled condition, whereas deliberately reversing the neural-to-acoustic mapping degraded perception. Future neuro-steered hearing studies should therefore quantify not only positive utility, such as intelligibility or preference gains, but also negative utility, including wrong-target exposure, instability burden, and adverse experiential cost.

This requirement is inseparable from population-specific evaluation. The intended user population for selective-hearing support is clinically heterogeneous, and future studies should report outcomes separately for subgroups defined at minimum by hearing-loss severity and age. Mild, moderate, and severe hearing loss should not be assumed to share the same translational priorities: some groups may benefit most from incremental speech-in-noise improvement, whereas others may be more sensitive to erroneous suppression, delayed switching, or excessive processing of already-audible speech. Likewise, older users may differ from younger users in calibration tolerance, sustained-use burden, attentional flexibility, and trust in adaptive device behavior. For this reason, future clinical evaluation should combine positive and negative outcome domains, including speech intelligibility or speech reception threshold, listening effort, fatigue, annoyance, discomfort, perceived control, trust, willingness to continue use, and subgroup-specific patterns of benefit and harm during prolonged listening.

Taken together, these priorities convert the major barriers to practical neuro-steered hearing into a coordinated experimental agenda. As summarized in Table 4, the field’s central task is not simply to optimize individual components, but to determine how neural representations, acoustic target formation, control policies, wearable sensing, and embedded implementation can jointly support reliable assistive behavior. This agenda provides a principled translational framework for linking neural inference to closed-loop device behavior and, ultimately, for evaluating whether neuro-steered control can deliver measurable and durable listening benefit.

## 5. Conclusions

Auditory attention decoding has reached an important turning point. The central question for the field is how neural inference can be translated into closed-loop control that improves listening in real-world multi-talker environments. In this context, the study by Choudhari et al. [14] provides a particularly important but bounded proof of principle: by linking real-time neural decoding to online gain control, it shows that attention decoding can provide a control principle for neuro-steered selective hearing, while leaving open whether this principle can be implemented with non-invasive sensing, wearable hardware, and clinically meaningful long-term benefit.

At the same time, this advance also clarifies what remains to be solved. The path toward practical non-invasive systems will depend on the joint success of decoder performance, low-latency control, robust candidate-signal formation in adverse acoustic scenes, wearable neural sensing, ecologically valid evaluation, and outcome measures that capture meaningful user benefit. It will also require principled integration between neural evidence and acoustic scene analysis, so that decoded attentional information can be translated into stable and perceptually useful control actions. From this perspective, the decisive issue is no longer the decoder in isolation, but the behavior of the complete loop: how neural evidence is formed, how acoustic targets are represented, how control actions are updated, and how these updates translate into durable listening benefit.

Closing the loop on the cocktail party effect will therefore require the field to determine when, how, and for whom closed-loop neural steering provides durable advantages over existing assistive strategies. If that shift can be achieved, auditory attention decoding may move beyond a compelling neuroscientific achievement and become a genuine foundation for the next generation of selective-hearing technologies.

## Figures and Tables

**Table 1:** Comparative translational characteristics of neural recording modes for neuro-steered selective hearing.

Neural Mode	Latency/Control Timescale	Accuracy/Control Reliability	Portability	Energy Consumption	Computational Complexity	Technological Maturity
Intracranial EEG/ECoG	Millisecond-scale neural recording; short-window real-time control is feasible in controlled settings.	High signal fidelity and high control reliability in proof-of-principle two-talker paradigms, but performance is participant- and implantation-dependent.	Very low for assistive translation; limited to implanted or clinical contexts.	Sensor acquisition may be manageable, but surgical implants, external hardware, and clinical infrastructure limit everyday use.	Moderate to high: participant-specific decoding, continuous neural inference, gain control, and safety monitoring.	Strong mechanistic and closed-loop proof of principle; low maturity as a scalable hearing-assistive platform.
Scalp EEG	Millisecond-scale acquisition, but reliable attention decisions often require multi-second evidence accumulation and smoothing.	Moderate and task-dependent; strongest in controlled two-speaker paradigms and more variable in realistic scenes or short windows.	Moderate: non-invasive and portable in laboratory/mobile setups, but conventional caps are not ideal for everyday hearing devices.	Low sensor power, but full closed-loop operation is constrained by battery life, wireless streaming, and embedded processing.	Moderate to high: artifact suppression, neural-acoustic matching, confidence estimation, temporal smoothing, and control updates.	Most plausible near-term non-invasive route; mature for laboratory auditory attention decoding, still emerging for wearable closed-loop devices.
Ear-centered/in-ear EEG	Millisecond-scale acquisition; short-window decisions are possible but often less reliable because of sparse channels and lower signal amplitude.	Low to moderate and highly montage-dependent; wearability improves while decoding performance may decrease relative to high-density scalp EEG.	High: compatible with unobtrusive, hearing-device-like form factors and prolonged use.	Low to moderate: favorable sensor energy profile, but contact-quality monitoring, wireless operation, and on-device processing add load.	Moderate: fewer channels reduce data volume, but myogenic artifacts, movement, impedance drift, and sparse-sensor decoding increase algorithmic burden.	Emerging translational platform; promising for everyday use but requires further validation of robustness, calibration burden, and long-term comfort.
MEG	Millisecond-scale recording with strong temporal resolution for resolving attention-related cortical dynamics.	High reliability for source-resolved laboratory analysis and demonstrated feasibility for second-scale probabilistic attention tracking. However, consistent improvements over EEG-based decoders and transfer to closed-loop hearing control have not been established.	Very low with conventional systems; wearable optically pumped magnetometer MEG is promising but not yet hearing-device-like.	Very high for conventional systems because of shielding, sensors, cooling or field-control infrastructure; lower but still substantial for emerging systems.	High: source modeling, interference suppression, and infrastructure-heavy acquisition.	Mature as a mechanistic and algorithm-development platform, with limited evidence of decoder-level translation. Cross-modality transfer to wearable EEG and device-level perceptual benefit remain at an early stage.
fNIRS	Slow hemodynamic response; suited to sustained attentional-state tracking over seconds rather than rapid talker switching.	Low to moderate for attention-state classification; useful for regional and sustained correlates but unlikely to support fast acoustic control alone.	Moderate to high: quiet, non-invasive, and more portable than fMRI, but head-mounted optical hardware remains visible and placement-sensitive.	Moderate: optical emitters and acquisition electronics require more power than passive electrophysiological sensing.	Low to moderate: fewer high-rate data streams than EEG/MEG, but motion correction and hemodynamic modeling are required.	Mature as a portable research tool; complementary validation modality rather than a primary near-term neuro-steering signal.
fMRI	Slow BOLD response and scanner constraints make it unsuitable for low-latency selective-hearing control.	High spatial specificity for localizing attentional modulation, but low practical control reliability for real-time hearing assistance.	None for everyday deployment; scanner-based and acoustically intrusive.	Very high because of scanner infrastructure, siting, and operation.	Very high: acquisition, preprocessing, artifact control, and whole-brain modeling are resource-intensive.	Highly mature for theory building and model constraints; not a translational platform for wearable closed-loop hearing.

**Table 2:** Concise comparison of EEG-based auditory-attention decoding methods for closed-loop selective hearing.

Decoder Family	Practical Conclusion	Recommended Closed-Loop Role
Canonical correlation analysis/regularized linear envelope decoders	Efficient, interpretable, and strong baselines when candidate speech envelopes are available; canonical correlation analysis improves stimulus–response correlation and classification sensitivity relative to conventional mappings [9,23].	Stream selection or gain updates after acoustic source separation.
Spatial filtering/beamforming-coupled auditory attention decoding	Improves neural signal-to-noise ratio and dimensionality reduction when spatial or stimulus structure is reliable; less effective when sources are co-located, moving, or poorly separated [24].	Beam steering or spatial source enhancement.
State-space/Bayesian tracking	Converts fluctuating short-window decisions into smoother probabilistic estimates; improves stability but introduces a controllable delay [12].	Anti-jitter switching and stable gain/beam updates.
Deep neural decoders	Can learn nonlinear spatiotemporal features and outperform linear baselines in selected auditory attention decoding tasks; higher data, training, and generalization demands [22,26].	Flexible stream, mask, or object-level control when deployment data match training conditions.

**Table 3:** Quantitative cross-comparison of conventional hearing technologies and neuro-steered selective hearing.

	Representative Quantitative Evidence and Current Strength	Main Limitation/Negative Finding
Conventional hearing-device acoustic path	Hearing-aid delay studies indicate that delays of about 20–30 ms can be disturbing; in open-fit conditions, 0.5–10 ms can affect preferred sound quality. Recent open-fit simulations showed unilateral SRT worsening from −18.1 to −14.7 dB signal-to-noise ratio across typical processing delays, with no comparable bilateral effect [37,38,39,40].	Defines a strict millisecond acoustic input-output budget, but it does not specify which talker the listener intends to follow; very low delay also limits computationally heavy enhancement.
Directional microphones and beamforming	Beamforming and gaze-directed beamforming can provide measurable speech-in-noise benefit when target direction and spatial separation are reliable; one head-mounted gaze/beamforming study reported practical benefit under favorable geometry [3,4,5,6,15,16].	Performance depends on reliable target direction, array geometry, and source separation; benefit can fall with moving talkers, non-frontal targets, reverberation, or spatially ambiguous scenes.
Noise reduction, speech enhancement, and speech separation	Traditional noise reduction often improves comfort or effort more consistently than intelligibility, whereas recent speech-enhancement and separation methods can improve SRT in controlled conditions [15,16,27,28,29,30].	Enhancement quality does not by itself solve target selection; deep models also introduce latency, compute, generalization, and artifact risks.
Gaze, head orientation, manual input, and audio-visual cue control	Gaze/head/manual/audio-visual systems provide fast observable intent cues; eye-gaze steering studies report improved speech intelligibility relative to no steering under controlled multi-talker conditions [3,4,5,6].	Overt behavior may diverge from covert listening intent during social gaze, monitoring, divided attention, or attention to a non-fixated talker.
Non-invasive scalp M/EEG AAD	Real-time Bayesian M/EEG tracking used about 1.5 s fixed-lag smoothing and approximately 1.75–1.9 s built-in delay; reported EEG switch performance was about 79.6% hit and 14.8% false alarm, whereas MEG switch performance dropped to about 64.1% hit and 26.2% false alarm [12].	Second-scale evidence accumulation creates a speed-stability trade-off; short decision windows and switch periods increase false-switch risk.
Wearable around-ear and in-ear EEG	Simultaneous comparisons report about 83.4% scalp EEG, 67.2% around-ear EEG, and 61.1% in-ear EEG accuracy on 60 s windows; dynamic ear-EEG work reports about 41% at 30 s and below 40% at 10 s in harder multi-talker settings [35,36].	Improved form factor comes with lower signal quality, stronger artifact sensitivity, sparse-channel constraints, and weaker short-window decoding.
Intracranial closed-loop AAD	The closed-loop system used a 4 s decoding window, 0.5 s updates, gain shifts up to ±9 dB target-to-masker ratio, and a mean instructed-switch time of 5.1 s; participant-level decoding accuracy ranged from 72.0% to 90.3% [14].	Evidence is invasive, sample-limited, scene-constrained, and seconds-scale; the hearing-loss validation used system-generated audio rather than online closed-loop neural control from hearing-impaired users.

**Table 4:** Practical roadmap for converting translational challenges into testable research tasks in neuro-steered selective hearing.

Translational Challenge	System-Level Design Recommendation	Evaluation Endpoints/Decision Criteria
Incremental and causally specific benefit of neural steering over strong non-neural baselines	Benchmark neural target selection as one control signal within a complete closed-loop pipeline. Keep the acoustic input, microphone configuration, enhancement back end, output-gain limits, and latency budget constant. Compare four control conditions: (1) acoustic-only selection; (2) non-neural intent cues, including eye gaze, head orientation, manual selection, and audio-visual cues; (3) neural target selection; and (4) oracle-target control. Where feasible, include counterbalanced condition order and fixed-gain, random-gain, reverse-gain, yoked-replay, or sham-control variants to separate online neural contingency from expectation, familiarization, adaptation to altered acoustics, and generic gain-modulation effects.	Closed-loop intelligibility or speech reception threshold; validated listening-effort ratings; response-time, task-accuracy, secondary-task, or pupillometry measures; user preference and everyday benefit; decoding accuracy; command-update delay; false-switch rate; recovery after erroneous updates; cumulative listening benefit; expectation or condition-belief ratings; order, familiarization, and adaptation effects.
Standardized testing paradigms, public datasets, and stratified user evaluation	Adopt a tiered benchmarking framework that combines offline public-dataset evaluation, replay-based closed-loop simulation, and online user testing. Use shared datasets to test neural decoding and cross-dataset generalization, while recognizing their limits for full closed-loop validation. Apply rigorous train-test separation, leave-one-trial/story/subject or cross-dataset validation, gaze-controlled testing where relevant, and matched acoustic backends across neural, acoustic-only, gaze-steered, multimodal, and oracle-control conditions. Stratify online evaluation by hearing status, age, device experience, sensor configuration, cognitive/working-memory profile where feasible, and calibration tolerance.	Cross-dataset generalization; condition-specific and participant-specific performance; decoding accuracy; effective switch time; false-switch, missed-switch, and recovery rates; speech intelligibility or speech reception threshold; validated listening-effort ratings; secondary-task, response-time, or pupillometry measures; user preference; calibration/recalibration time; comfort and skin tolerance; cross-session stability; everyday user-reported benefit in normal-hearing, hearing-impaired, and wearable-use subgroups.
Neural-acoustic target representation and candidate formation	Move from predefined two-talker streams to scene-derived candidate targets. In matched acoustic scenes, compare stream/envelope, spatial-object, spectrotemporal-mask, and semantic or multimodal scene hypotheses produced by source separation, beamforming, multi-channel Wiener filtering, or pretrained audio/audio-visual encoders. Require each candidate representation to be both neurally testable and mappable onto gain, beam, or mask control.	Neural-acoustic correspondence; target-object continuity; source/object tracking stability; separation, beamforming, or mask robustness under noise, reverberation, listener or talker movement, and competing talkers; post-enhancement intelligibility, speech reception threshold, listening effort, and user preference.
Low-latency closed-loop architecture	Specify separate timing budgets for the acoustic input-output path and the neural-control update path. The acoustic rendering path should remain within hearing-device delay constraints, whereas neural steering should be treated as asynchronous updating of gain, mask, or beam parameters rather than frame-by-frame waveform control. Report the neural evidence window, update interval, fixed-lag or smoothing delay, computation time, and effective attention-to-control switch time.	Acoustic input-output delay; neural evidence window; update interval; computational latency; fixed-lag or temporal-smoothing delay; effective attention-to-control switch time; latency-benefit trade-off; false-switch rate; embedded power and thermal cost.
Uncertainty-aware strategy control	Introduce a strategy-control layer that converts probabilistic neural-acoustic matching into bounded and reversible acoustic interventions. Use confidence calibration, thresholding or hysteresis, temporal evidence integration, state persistence, and fallback or temporary suspension of neural steering when neural or acoustic evidence is unreliable.	Confidence calibration; false intervention and missed-switch rates; control over-switching or under-switching; perceptual discontinuity; recovery time after incorrect updates; stability during endogenous attention switches, scene changes, and low-confidence periods; graceful degradation relative to an acoustic-only fallback.
Wearable EEG, personalization, and long-term robustness	Treat scalp, around-ear, and in-ear EEG translation as sensor-algorithm-control co-design rather than decoder portability alone. Combine stable ear-centered or auricular-conformal interfaces, high-input-impedance active electrodes, contact-quality or impedance monitoring, auxiliary motion/noise/EMG references, real-time artifact suppression, subject-adaptive decoding, online self-supervised updating, and event-triggered recalibration.	Cross-session closed-loop benefit; calibration and recalibration time; placement, montage, and impedance sensitivity; robustness to head movement, jaw motion, muscle artifacts, and contact drift; comfort and skin tolerance during prolonged use; performance in hearing-impaired listeners under ecologically valid listening conditions.
Embedded and device-level feasibility	Tie real-time feasibility claims to a declared hardware platform and processing target. Budget computation for multi-microphone capture, scene analysis, candidate-signal formation, EEG preprocessing, neural-acoustic matching, confidence estimation, and control updating. Use compact representations, recursive or adaptive decoders, low-complexity candidate generation, compression, quantization, distillation, cascaded or event-triggered inference, sparse or approximate computation, and hearing-device/companion-device distribution when needed.	End-to-end computation latency; memory footprint and data movement; numerical precision effects; processor load; power consumption and battery life; skin-facing temperature and thermal comfort; sustained operation; reliability under prolonged real-time use.
Foundation-model and multimodal integration	Use pretrained audio, audio-visual, audio-language, and EEG models to generate richer scene hypotheses, reduce calibration burden, or support user interaction only when their outputs remain neurally testable and mappable onto controllable streams, masks, spatial targets, or longer-timescale control states. Benchmark foundation-model-assisted pipelines against classical pipelines under the same latency, privacy, controllability, and embedded-resource constraints.	Scene-understanding gain; candidate controllability; calibration reduction; cross-session and cross-user generalization; robustness in natural conversation; privacy and hallucination risk; computational cost; closed-loop benefit relative to classical auditory-attention-decoding and acoustic-only pipelines.
Clinical, regulatory, and human-factors readiness	Treat clinical/regulatory translation as an upstream design constraint rather than a final administrative step. Define intended users, use environments, and foreseeable failure modes; document risk controls for erroneous target selection, unsafe or disruptive gain/beam/mask updates, thermal exposure, skin-contact problems, privacy-sensitive neural data, software updates, and companion-device dependence; and include human-factors/usability testing together with clinically meaningful outcome validation.	Risk-control verification; acoustic and thermal safety; skin-contact tolerance; usability, fitting, and training time; data privacy and cybersecurity; software-update traceability; adverse-event and failure-mode monitoring; durable speech-in-noise, listening-effort, and everyday-benefit outcomes.

## Data Availability

No new data were created or analyzed in this study. Data sharing is not applicable to this article.
